# Sonication fluid cultures enhance pathogen identification in fracture-related-infection (FRI)

**DOI:** 10.5194/jbji-11-431-2026

**Published:** 2026-07-20

**Authors:** Anas Zouitni, Erlangga Yusuf, Jakob van Oldenrijk, Tjebbe Hagenaars, Peter D. Croughs, P. Koen Bos, Denise Eygendaal, Ewout S. Veltman

**Affiliations:** 1 Department of Orthopaedic Surgery and Sports Medicine, Erasmus Medical Center, dr. Molewaterplein 40, 3015 GD Rotterdam, the Netherlands; 2 Department of Medical Microbiology and Infectious Diseases, Erasmus Medical Center, dr. Molewaterplein 40, 3015 GD Rotterdam, the Netherlands; 3 Trauma Research Unit, Department of Surgery, Erasmus Medical Center, dr. Molewaterplein 40, 3015 GD Rotterdam, the Netherlands

## Abstract

**Background**: Sonication fluid culture (SFC) of osteosynthesis material may be a valuable adjunct tool to diagnose fracture-related infection (FRI). This study aims to evaluate the added diagnostic value of SFC as a diagnostic tool. The diagnostic value of SFC was assessed by evaluating its impact on confirming microbiological results and changing diagnosis. **Methods**: We analysed patients undergoing osteosynthesis hardware removal between 2012 and 2021. We categorized patients using FRI consensus criteria: suspected FRI or confirmed FRI. The sensitivity and specificity of tissue cultures and SFC were assessed. The added value of SFC was determined as the number of cases where SFC was essential for confirming FRI and the influence on antibiotic therapy selection. **Results**: We included 96 patients with three or more tissue cultures. Based on diagnosis without SFC, we found 14 aseptic, 35 suggestive FRI cases, and 47 confirmed FRI cases. Following SFC results, four cases changed diagnosis from aseptic to suggestive FRI, and three cases changed from suggestive FRI to confirmed FRI. The sensitivity of tissue culture was 74 %, and the specificity was 98 %. The sensitivity of SFC was 84 %, and the specificity was 83 %. Combining tissue cultures with SFC significantly increased sensitivity compared with tissue cultures alone, from 74 % to 88 % ( p<0.001 ). In 21 confirmed FRIs (42 %), SFC results were concordant with tissue cultures and would not alter FRI management. SFC detected an additional virulent pathogen in three confirmed FRIs (6 %) and low-virulent pathogens in eight confirmed FRIs (16 %) which could influence the antibiotic regimen.

**Conclusions:** SFC plays a significant role in diagnosing FRI by enhancing pathogen detection. SFC may influence FRI management in approximately 6 % to 16 % of confirmed FRI. In every FRI, an adequate number of cultures according to protocol should be obtained, and SFC should also be performed.

## 1 Introduction

Fracture-related infection (FRI) is a serious complication after fracture fixation (Bezstarosti et al., 2019). Reported infection rates range from 1 % to 2 % in closed fractures to 29 % in open fractures (Natoli et al., 2025; Walter et al., 2021). Diagnosing FRI remains complex due to the variety of symptoms. The international fracture-related infection (FRI) group consensually defined FRI using clinical, laboratory, and imaging criteria (Metsemakers et al., 2018).

Conventional culture methods are often negative or inconclusive. Literature reports 9 % to 22 % of confirmed FRIs with negative bacterial cultures (Pilskog et al., 2023; Gitajn et al., 2016). Sonication is a method using high-frequency sound waves to generate powerful forces (Trampuz et al., 2003). The implant is placed in a sonication bath transmitting ultrasound, releasing bacteria from the biofilm. Literature indicates polymicrobial infections in up to 30 % of FRIs (Kuehl et al., 2019; Yano et al., 2014). Sonication fluid culture (SFC) detects more polymicrobial infections compared to tissue cultures (Yano et al., 2014). SFC yields results faster than conventional cultures, showing results within 24 h in over 85 % cases (Henssler et al., 2024). Empirical antimicrobial treatment has a reported 50 % mismatch rate for targeted antibiotics (Jacobs et al., 2024). SFC could help in identifying all pathogens, and fast results may allow an earlier transition to targeted therapy.

SFC has not been widely adopted due to concerns about added diagnostic value, accuracy, labour intensity, and costs. Multiple studies show SFC to be of great value in periprosthetic joint infection (PJI) diagnostics, demonstrating higher sensitivity than conventional tissue cultures (Watanabe et al., 2024). We recently evaluated the added diagnostic value of routine SFC in revision hip or knee arthroplasty. In a large proportion of PJI (11 %), SFC results were essential for diagnosis (Zouitni et al., 2024). In approximately 8 % of PJI, SFC detected an additional virulent microorganism that could influence antibiotic therapy selection (Zouitni et al., 2025). SFC is considered to be an adjunct tool, relying on tissue cultures to meet the criteria for confirming FRI (Metsemakers et al., 2018). Most studies on FRI lack a clear consensus definition, have small cohorts, and do not evaluate how SFC influences diagnosis (Finelli et al., 2021; Onsea et al., 2018).

This study aims to evaluate the added diagnostic value of SFC for FRI diagnosis. We will determine how often SFC is decisive for confirming FRI in addition to conventional diagnostic methods. We hypothesize that SFC improves diagnostic sensitivity and specificity for FRI when used alongside conventional tissue cultures.

## 2 Materials and methods

### 2.1 Study population

We reviewed prospectively collected data from patients who underwent partial or complete removal of osteosynthesis implants between July 2012 to May 2021 at a tertiary university hospital. We sent the retrieved implants to our microbiology lab for SFC. Reasons for implant removal include both septic and aseptic causes. We aimed to send all removed osteosynthesis material for SFC, also without suspicion of infection. The minimum diagnostic workup was that all patients received examinations for clinical signs of infection (e.g. wound defects, sinus tracts) and radiological imaging. If the diagnosis was unclear or if there was suspicion of infection, more diagnostic methods were used, such as serum inflammatory markers and blood cultures. Histopathological examination was not part of routine diagnostics. Patients who had fewer than three tissue cultures obtained were excluded from the analysis.

We classified patients based on the consensus FRI criteria defined by the AO foundation and the EBJIS, fused as the FRI group (Metsemakers et al., 2018). Patients were classified as “suggestive FRI” or “confirmed FRI”. Although not included in the consensus definition, we classified patients as “aseptic” if they did not meet any FRI criteria. Patients were classified before and after incorporating SFC results to evaluate if these results led to a new confirmed FRI. We analysed FRI cases based on the time interval between the index surgery and onset of symptoms: early (less than 2 weeks), delayed (2–10 weeks), and late FRI (more than 10 weeks) (Stevenson et al., 2022).

Added diagnostic value was defined as the number of patients for whom SFC was essential for pathogen detection and confirming diagnosis. SFC was considered to be essential for pathogen identification when tissue cultures alone yielded inconclusive results. Additionally, in patients with (preoperative) confirmed FRI by non-microbiological criteria, a positive SFC was considered to have added value when tissue cultures yielded a single positive result.

Patients received empiric antibiotic treatment in the case of high suspicion of infection or sepsis. Based on definite culture results, antibiotic treatment was tailored to targeted therapy or discontinued if negative, a decision made in close consultation with the medical microbiologist.

### 2.2 Perioperative cultures

We aimed to obtain at least three tissue cultures from every patient. All tissue cultures were taken with separate sterile rongeurs and placed in separate sterile containers. The containers were immediately delivered to the microbiology laboratory for further analysis. Tissue samples were divided into 0.1 mL aliquots and placed onto blood agar, chocolate agar, McConkey agar, and Brucella blood agar. The cultures were incubated at 35–37 °C for 14 d. Blood, chocolate, and McConkey plates were incubated in CO_2_ 5 %, and the Brucella blood agar plate was incubated anaerobically.

### 2.3 SFC

The osteosyntheses components that were removed during surgery were sent to the microbiology laboratory in a sterile hard-polypropylene container. Material was processed within 6 h after implant removal. A sodium chloride solution was added to cover at least 90 % of the implant. The container was placed in an SFC bath (BactoSonic 14.2, Bandelin electronic, Berlin, Germany) for 1 min at 40 kHz. Before and after sonication, the container was vortexed manually for 30 s. Subsequently, 100 µ L of SFC fluid was transferred to blood, chocolate, and Brucella agar plates, and 10 mL was inoculated into aerobe and anaerobe blood culture bottles. All media were incubated at 35–37 °C for 14 d, with blood and chocolate agar plates being incubated in 5 % CO_2_, and the Brucella agar was incubated anaerobically. In case of growth on the plates, the number of colony-forming units was counted. Microorganisms were classified by using the matrix-assisted laser desorption/ionization time-of-flight analyser (MALDI-ToF) system (BrukerDaltonics). According to FRI criteria, SFC as separate diagnostics cannot confirm infection, and no cut-off is given as a diagnostic threshold. Differentiation between possible contamination or true pathogens was therefore determined together with other diagnostic criteria and consensus during multidisciplinary team meetings.

### 2.4 Microbiology

Microorganisms found by tissue and SFC were analysed. According to FRI consensus criteria, pathogens are confirmed when at least two separate deep tissue and/or implant cultures (including SFC) yield the same microorganism. No diagnostic cut-off value for a positive SFC is defined by these criteria.

A contaminant is a microorganism that is unlikely to be causative for infection and more likely to have been introduced during sample collection, processing, or analysis. Single positive cultures were considered to be suggestive of infection and interpreted alongside clinical findings. In case (highly) virulent pathogens are identified, one positive sample increases suspicion of infection (Depypere et al., 2020). We considered the following to be virulent microorganisms: *Staphylococcus aureus*, *Staphylococcus lugdunensis*, *Enterococcus* spp., *beta-hemolytic streptococci*, *Enterobacterales*, and *Pseudomonas aeruginosa* (Ryan, 2022). *Cutibacterium acnes*, *Coagulase-negative Staphylococci*, *Corynebacterium* spp., anaerobes, and other microorganisms not commonly associated with FRI were considered to be low-virulence microorganisms for the purpose of this study. *Micrococcus* spp. was considered to be a contaminant. All culture results were discussed in multidisciplinary team meetings, including our microbiology department, to establish diagnosis and treatment plans.

### 2.5 Statistical analysis

We summarized patient characteristics using descriptive statistics. Percentages were used for presenting categorical variables. Descriptive comparisons between the categorical variables were performed using Pearson's $\chi^{2}$ test or Fisher's exact test. The sensitivity, specificity, negative predictive value, and positive predictive value of the diagnostic tools were calculated using 2×2 contingency tables. We used FRI consensus criteria as a reference standard to determine these values (Metsemakers et al., 2018). We calculated the combined sensitivity and specificity of SFC and tissue culture by defining a positive result as the isolation of identical organisms from tissue cultures or concordant growth of identical organisms in a single tissue culture and SFC. Factors associated with FRI were assessed for the relationship between positive SFC by using univariate analysis and multivariate logistic regression analysis. Variables were selected based on clinical relevance of implant-related factors for inclusion in multivariate analysis. A p value below 0.05 for a two-sided test was considered to be statistically significant. We performed the statistical analysis using IBM SPSS Statistics (version 28.0.1.0).

## 3 Results

### 3.1 FRI cases

We analysed 96 patients with three or more tissue cultures and SFC. Postoperative diagnosis based on FRI criteria (without SFC), included 14 aseptic cases, 35 suggestive FRIs, and 47 confirmed FRIs (Table 1).

**Table 1:** Case features (postoperative diagnosis, before including SFC).

	Aseptic	Suggestive	Confirmed
	( N=14 )	FRI	FRI
		( N=35 )	( N=47 )
Characteristic			
Age (years), median (range)	63 (12–80)	61 (9–84)	54 (11–86)
Sex (Male, % of total)	9 (64 %)	13 (37 %)	30 (64 %)
Fracture location			
Humerus	0	2 (6 %)	1 (2 %)
Ulna, radius, hand	0	1 (3 %)	4 (9 %)
Pelvis, femur, patella	9 (64 %)	23 (66 %)	19 (40 %)
Tibia, fibula, foot	5 (36 %)	9 (26 %)	21 (45 %)
Other (cranium, maxillofacial)	0	0	2 (4 %)
Open fractures	0	5 (14 %)	10 (21 %)
Implant type			
Plates and screws	7 (50 %)	19 (54 %)	19 (40 %)
Only screw(s)	4 (29 %)	11 (31 %)	18 (38 %)
Dynamic/canulated hip screws	3 (21 %)	5 (14 %)	6 (13 %)
Other (cerclage wires, Kirschner wires)	0	0	4 (11 %)
ASA score			
ASA 1	2 (14 %)	4 (11 %)	10 (21 %)
ASA 2	7 (50 %)	20 (57 %)	23 (49 %)
ASA 3 +	5 (36 %)	11 (31 %)	14 (30 %)
Osteosynthesis material removal			
Complete removal	6 (43 %)	7 (20 %)	30 (64 %)
Partial removal	1 (7 %)	2 (6 %)	6 (13 %)
Exchange	7 (50 %)	26 (74 %)	11 (23 %)
(Pre-operative) confirmed criteria			
Fistula or sinus tract	0	0	24 (51 %)
Wound breakdown^a^	0	0	6 (13 %)
Purulent drainage from the wound	0	0	19 (40 %)
Presence of pus during surgery	0	0	22 (47 %)
(Pre-operative) suggestive criteria			
Pain^b^	0	4 (11 %)	16 (34 %)
Local redness	0	2 (6 %)	19 (40 %)
Local swelling	0	4 (11 %)	22 (47 %)
Increased local temperature	0	0	8 (17 %)
Fever ( ≥38.3 °C (101 °F))	0	1 (3 %)	1 (2 %)
Persistent wound drainage^c^	0	4 (11 %)	19 (40 %)
Bone lysis or implant loosening (radiological)	0	9 (26 %)	11 (23 %)
Non-union (radiological)	0	28 (80 %)	11 (23 %)
Elevated serum inflammatory markers^d^			
Erythrocyte sedimentation rate (ESR), ≥ 30 mm h^−1^	0 / 9	10 / 29 (34 %)	17 / 32 (53 %)
C-reactive protein (CRP), ≥10 mg L^−1^	0 / 10	10 / 31 (32 %)	12 / 42 (29 %)
White blood cell count (WBC), $>10\times 10^{9}$ L	0 / 11	4 / 31 (13 %)	3 / 44 (7 %)
Number of intraoperative tissue cultures			
Three specimens	11 (79 %)	11 (31 %)	20 (43 %)
Four specimens	1 (7 %)	7 (20 %)	9 (19 %)
Five or more specimens	2 (14 %)	17 (49 %)	18 (38 %)

^a^ With communication to the bone or implant, visualization of the material. ^b^ Without weight bearing, increasing over time, new onset. ^c^ Increasing or new onset wound drainage, beyond the first few days postoperatively, without solid alternative explanation. ^d^ Included as suggestive signs in case of a secondary rise (after an initial decrease) or a consistent elevation over a period in time and after exclusion of other infectious foci or inflammatory processes.

### 3.2 Added diagnostic value of SFC

**Figure 1:**
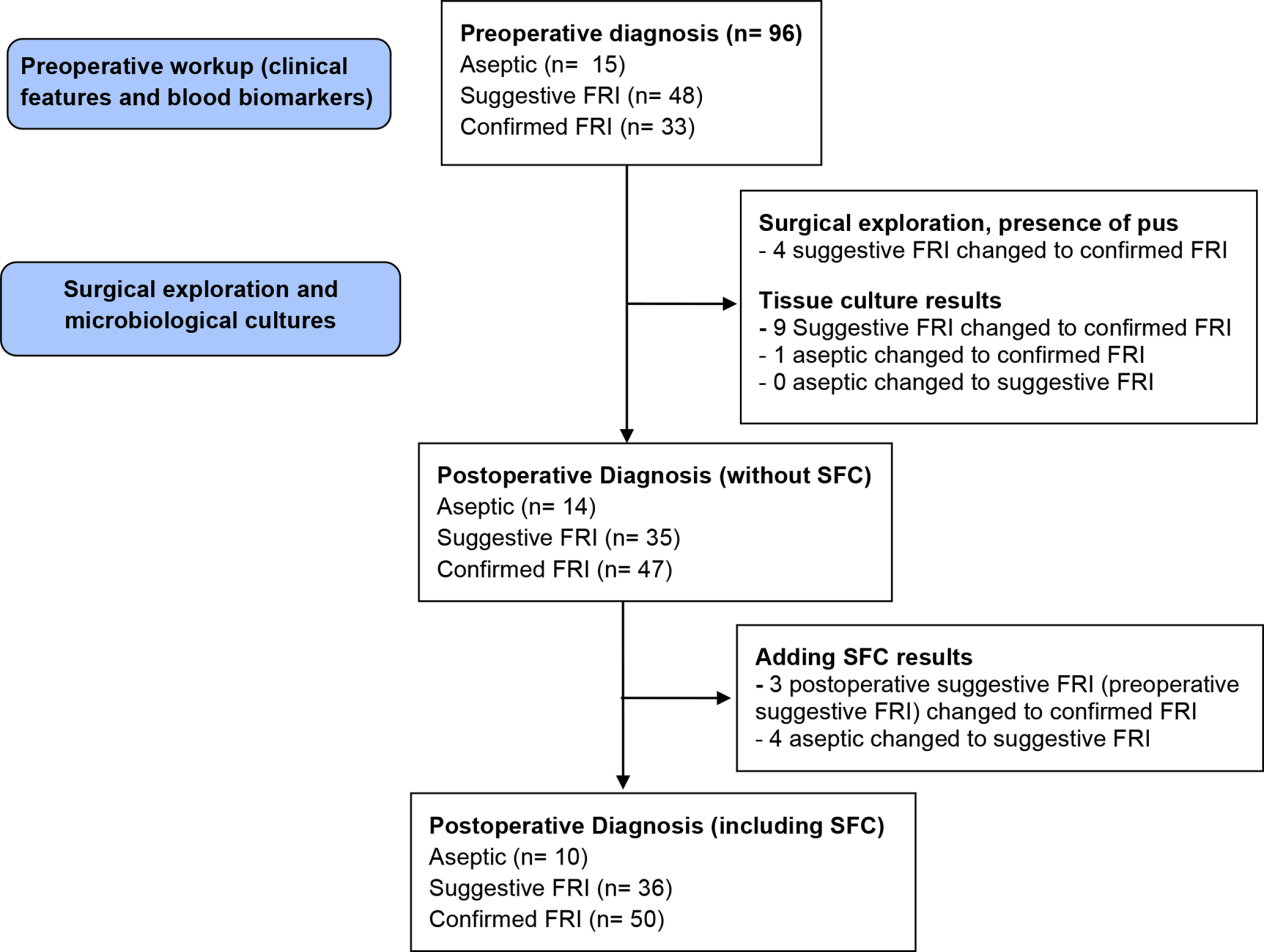
FRI diagnosis flow diagram.

After including SFC results, four aseptic cases changed diagnosis to suggestive FRI, and three cases changed from suggestive FRI to confirmed FRI (Fig. 1). The three cases that changed to confirmed FRI included two late-onset FRIs and one early-onset FRI.

In 37 confirmed FRIs (74 %), the diagnosis was initially confirmed using non-microbiological criteria (Fig. 1). In three of these cases (8 %), SFC was needed to achieve microbiological confirmation together with a single positive tissue culture.

In 18 / 50 confirmed FRIs (36 %), five or more tissue cultures were obtained. In these cases, SFC was not essential for confirming FRI.

### 3.3 Sensitivity and specificity

The sensitivity of tissue culture was 74 %, and the specificity was 98 % (Table 2). The sensitivity of SFC was 84 %, and the specificity was 83 %. Tissue culture had lower sensitivity compared to SFC (74 % vs. 84 %, with p<0.002 ). Tissue cultures had higher specificity compared to SFC cultures, but this difference was not statistically significant (98 % vs. 83 %, p<0.174 ). The sensitivity of tissue cultures alone was significantly lower than tissue cultures combined with SFC (74 % vs. 88 %, with p<0.001 ). Specificity of tissue cultures alone was significantly higher than the combination of tissue cultures with SFC (98 % vs. 83 %, with p<0.001 ).

**Table 2:** Sensitivity, specificity, positive predictive value, and negative predictive value.

Method	Confirmed	Not	Sensitivity	Specificity	Positive	Negative
	FRI	confirmed	% (95 % CI)	% (95 % CI)	predictive	predictive
	( n=50 )	( n=46 )			value	value
					% (95 % CI)	% (95 % CI)
TC^*^ positive	37	1	74 % (61 %–85 %)	98 % (91 %–100 %)	97 % (89 %–100 %)	78 % (66 %–87 %)
SFC^*^ positive	42	8	84 % (72 %–92 %)	83 % (70 %–92 %)	84 % (72 %–92 %)	83 % (70 %–90 %)
TC^*^ + SFC positive	44	8	88 % (77 %–95 %)	83 % (70 %–92 %)	85 % (73 %–93 %)	86 % (74 %–94 %)

^*^ TC denotes tissue cultures, and SFC denotes sonication fluid culture.

### 3.4 Detected microorganisms

The most common identified microorganisms were *Staphylococcus aureus,* *Cutibacterium acnes*, and *Staphylococcus epidermidis* (CoNS) (Table 3). Based on preoperative diagnosis, SFC was more frequently positive in all diagnostic groups. Tissue cultures were positive in 45 cases: 1 / 15 aseptic, 16 / 48 suggestive FRIs, and 28 / 33 confirmed FRIs. SFC was positive in 54 cases: 5 / 15 aseptic, 20 / 48 suggestive FRIs, and 29 / 33 confirmed FRIs. Based on postoperative diagnosis, tissue cultures yielded polymicrobial results in 22 / 50 confirmed FRIs (44 %), and SFC yielded results in 20 / 50 confirmed FRIs (40 %). SFC yielded isolated positive results in four aseptic cases, four suggestive FRI cases, and three confirmed FRI cases.

**Table 3:** Microorganisms identified in postoperative confirmed FRIs ( n=50 ).

Microorganisms	TC^*^	SFC^*^	Identical
			microorganisms
			found by TC^*^
			and SFC^*^
Staphylococci			
*Staphylococcus aureus*	19	19	18
Coagulase-negative Staphylococci (CoNS)			
*Staphylococcus epidermidis*	5	8	5
*Staphylococcus capitis*	3	5	3
*Staphylococcus hominis*	1	0	0
*Staphylococcus lugdunensis*	1	1	1
Staphylococcus species	1	1	0
*Staphylococcus schleiferi*	0	1	0
Streptococci			
*Streptococcus oralis* (Str. mitis-group)	1	3	1
*Streptococcus agalactiae* (hemolytic Streptococci group B)	1	1	1
*Streptococcus parauberis*	0	1	0
Enterococci			
*Enterococcus faecium*	2	3	2
*Enterococcus faecalis*	2	4	1
*Enterococcus gallinarium*	0	1	0
Enterobacteriaceae			
*Escherichia coli*	5	5	5
*Enterobacter cloacae* complex	4	1	1
*Proteus vulgaris*	2	2	2
*Serratia marcescens*	1	0	0
*Klebsiella oxytoca*	1	1	1
*Morganella morganii*	1	1	1
*Proteus mirabilis*	1	1	1
Corynebacteria	4	1	0
Other			
*Cutibacterium acnes*	8	6	4
*Pseudomonas aeruginosa*	1	2	0
*Finegoldia magna*	2	0	0
*Micrococcus luteus*	0	2	0
*Bacteroides fragilis*	1	0	0
*Propionibacterium avidum*	2	2	2
*Eikenella corodens*	0	1	0
*Fusobacterium nucleatum*	2	2	2
Acetinobacter species	0	1	0
*Bacillus megaterium*	0	1	0

^*^ TC denotes tissue cultures, and SFC denotes sonication fluid culture.

### 3.5 Preoperative antibiotics

A total of 12 cases received antibiotics between 1 and 14 d before removal of hardware (2 suggestive FRIs, 10 confirmed FRIs). Tissue cultures were positive in 8 / 12 cases (67 %), and SFC was positive in 10 / 12 cases (83 %). This included two cases where SFC was positive with negative tissue cultures and three cases with positive SFC and one positive tissue culture.

In one suggestive FRI, SFC confirmed FRI together with tissue culture by identifying a virulent pathogen (*Pseudomonas aeruginosa*). In one confirmed FRI, SFC was isolated as positive and identified *Staphylococcus aureus* with negative tissue cultures.

### 3.6 Postoperative antibiotic therapy

A total of 51 cases were treated as FRIs with antibiotic therapy, including 49 / 50 confirmed FRIs and 2 / 36 suggestive FRIs. In one confirmed FRI, no antibiotic therapy was given, and an observational (watchful waiting) management approach was implemented. Cases that were reclassified from aseptic to suggestive FRI did not receive antibiotic therapy.

In 37 / 50 confirmed FRI cases, tissue cultures confirmed infection by identifying identical pathogens in at least two specimens. In 21 / 50 (42 %), SFC would not have altered antibiotic therapy as the pathogens were concordantly identified by tissue culture. In 13 / 37 cases, SFC additionally detected a virulent pathogen not identified by tissue cultures (all *Enterococcus* spp.), which influenced the antibiotic therapy regimen. In 10 / 13 cases, SFC identified additional low-virulent pathogens not detected by tissue culture, which could have influenced antibiotic therapy in eight cases (16 %). *Micrococcus luteus* was additionally detected in two cases and was considered to be a contaminant.

### 3.7 Factors associated with positive SFC

SFC was positive in 54 cases (Table 4). Of these, six occurred in delayed FRIs (implant removal 2–10 weeks after surgery), and 48 occurred in late FRIs ( >10 weeks). Although not statistically significant, SFC was more frequently positive in patients with a history of open fractures (9 / 15 cases; p=0.750 ). In cases with implant loosening, SFC was positive in 15 / 20 cases ( p=0.057 ). Non-union was significantly associated with negative SFC results (24 / 39; p=0.004 ). Among patients with preoperative antibiotic therapy, SFC was significantly associated with a positive result ( p=0.031 ).

**Table 4:** Implant-related variables with positive or negative SFC.

Variables/categories	Positive	Negative	p value^a^
	SFC	SFC	
	( n=54 )	( n=42 )	
Time to removal			
<2 weeks	0	0	
2–10 weeks	6 / 9	3 / 9	0.508
>10 weeks	28 / 87	39 / 87	0.508
Fracture location			
Humerus	1 / 3	2 / 3	0.579^b^
Ulna, radius, hand	5 / 5	0 / 5	0.066^b^
Pelvis, femur, patella	23 / 51	28 / 51	0.019
Tibia, fibula, foot	23 / 35	12 / 35	0.157
Other (cranium, maxillofacial)	2 / 2	0 / 2	0.503^b^
Open fracture	9 / 15	6 / 15	0.750
Implant type			
Plates and screws	26 / 45	19 / 45	0.777
Only screw(s)	19 / 33	14 / 33	0.850
Dynamic/canulated hip screws	6 / 14	8 / 14	0.274
Other (cerclage wires, Kirschner wires)	3 / 4	1 / 4	0.629^b^
Radiology			
Bone lysis or implant loosening	15 / 20	5 / 20	0.057
Non-union	15 / 39	24 / 39	0.004
Non-microbiological confirmed FRI criteria			
Fistula or sinus tract	23 / 24	1 / 24	<0.001
Wound breakdown with visualization of hardware	5 / 6	1 / 6	0.226^b^
Purulent drainage from the wound^c^	17 / 19	2 / 19	0.001
Presence of pus during surgery	20 / 22	2 / 22	<0.001
Preoperative antibiotics	10 / 12	2 / 12	0.043

^a^ All variables calculated with $\chi^{2}$ test, except for ^b^ Fisher's exact test. ^*c*^ Increasing or new onset wound drainage, beyond the first few days postoperatively, without solid alternative explanation.

In binary logistic regression analysis, non-union was independently associated with lower odds of positive SFC ([OR] 0.3, 95 % CI: 0.1–0.7, with p=0.005 ) (Table 5). Implant loosening was associated with higher odds of positive SFC ([OR] 3.8, 95 % CI 1.1–12.4, with p=0.034 ).

**Table 5:** Variable association with positive SFC (binary logistic regression).

Factor	Odds	95 % CI	p value
	ratio		
Open fracture	1.2	0.3–3.6	0.989
Preoperative antibiotics	6.1	0.6–17.1	0.151
Non-union	0.3	0.1–0.7	0.005
Implant loosening	3.8	1.1–12.4	0.034

## 4 Discussion

This is the first study to assess the added diagnostic value of SFC in FRI diagnostics using prospectively collected data. Our findings demonstrate that SFC is essential in identifying pathogens and confirming diagnosis in 6 % of confirmed FRIs. Without SFC, 6 % to 16 % of confirmed FRIs would receive suboptimal treatment because not all pathogens would be detected. This finding aligns with our previous work on periprosthetic joint infection (PJI), where SFC influenced antibiotic therapy in 8 % to 20 % of confirmed PJIs (Zouitni et al., 2025).

Combining tissue cultures with SFC significantly increased sensitivity from 74 % to 88 %, although it reduced specificity. Reported sensitivity and specificity vary widely in literature, with sensitivity ranging from 57 % to 100 % for tissue cultures and 87 % to 100 % for SFC. Specificity ranges from 89 % to 100 % for tissue cultures and 50 % to 100 % for SFC (Onsea et al., 2018; Velasquez et al., 2024; Bellova et al., 2021; Finelli et al., 2021). The heterogenous results in previous studies provide limited clarity on the diagnostic accuracy of SFC. Differences in cohort characteristics, the use of FRI consensus criteria, and culture methods could explain the observed heterogeneity. Studies that include the FRI consensus criteria and used SFC inoculated blood culture bottles report superior sensitivity compared to tissue cultures. The combination of tissue cultures and SFC has been shown to increase sensitivity for FRI (Ueda et al., 2019). In PJI literature, combining tissue culture and SFC has been shown to increase sensitivity without compromising the specificity.

SFC appeared to be more sensitive than tissue cultures in patients who received preoperative antibiotics. Tissue cultures were sufficiently positive in 5 / 12 cases (42 %), whereas SFC was positive in 10 / 12 cases (84 %), suggesting added value when tissue cultures show a single positive result. In PJI literature, it is suggested that SFC has a trend toward higher sensitivity and specificity than tissue cultures in patients who received antibiotics preoperatively. Studies on FRI show similar results, but most studies have small sample sizes and do not assess osteosynthesis hardware separately from prosthetic joint material (Onsea et al., 2018).

Microbiological confirmation is of great importance in FRI diagnosis and treatment. In our cohort, 26 % of confirmed FRIs did not meet non-microbiological confirmed FRI criteria. A similar finding has been reported previously, with clinical confirmatory criteria absent in 23 % of FRIs (Vanvelk et al., 2023). This highlights the importance of optimizing microbiologic sampling by obtaining enough tissue cultures and SFC to minimize diagnostic uncertainty. According to the FRI consensus criteria, three or more tissue cultures should be obtained intraoperatively (Metsemakers et al., 2018). Our findings demonstrate that, even when this threshold is met, SFC is essential for pathogen identification in 6 % of confirmed FRI. More recent recommendations of the FRI consensus group advise obtaining at least five tissue cultures (Govaert et al., 2020). In our cohort, approximately 36 % of confirmed FRI cases met this threshold. In these cases, SFC contributed to increasing the microbiological yield, but it was not essential for confirming FRI. Future studies should routinely obtain this recommended number of tissue cultures when evaluating the added value of SFC.

SFC found relatively fewer polymicrobial results compared to tissue cultures (40 % vs. 44 %, respectively). This finding differs from an earlier study where SFC detected more polymicrobial infections than tissue cultures (Yano et al., 2014). SFC as an additional tool may be helpful in identifying all causative pathogens for polymicrobial FRI.

The clinical relevance of an isolated positive SFC for FRI remains uncertain. In contrast to PJI, interpretation of an isolated SFC is more complex because it cannot independently confirm diagnosis (Metsemakers et al., 2018). A validation study of the FRI definition criteria found that infection is highly likely in the case of a single positive culture with a virulent pathogen (Onsea et al., 2022). A study on PJI showed that an isolated positive SFC was almost twice as frequent in PJI during follow-up; however, this finding could also represent a new infection (Rondaan et al., 2023). A study on FRI reported that, in 5 % of FRIs without clinical confirmatory criteria, a single positive culture with a virulent organism was found (Vanvelk et al., 2023). This finding was not evaluated for an isolated SFC. In our cohort, isolated positive SFC results were observed in all diagnostic groups. All aseptic or suggestive FRIs with an isolated positive SFC included low-virulent microorganisms.

The limitations of our study include the fact that SFC was not routinely performed in all cases in our hospital, leading to potential selection bias. The early inclusion period could explain the inconsistency of diagnostics as the FRI definition is relatively new.

The small sample size limits subgroup analyses, particularly regarding the interpretation of isolated positive SFC findings and its role in presumed aseptic cases. We found that 33 % of aseptic patients showed a positive SFC. Other studies show that SFC is positive in up to 27 % of patients without clinical signs of infection (Fuchs et al., 2019; Palmowski et al., 2021). In our study, contamination is likely an explanation for this finding due to the additional handling of the culture process. The pathogens identified were consequently low-virulent microorganisms (Coagulase-negative *Staphylococci* (four cases) and *Cutibacterium acnes* (one case) with low microbial yield (10 CFU per mL).

## 5 Conclusion

SFC plays a significant role in diagnosing and treating FRI by enhancing pathogen detection. Combining tissue cultures with SFC improves diagnostic sensitivity and specificity. These findings support the routine use of SFC in FRI diagnostics. Exchanging a single screw for SFC culture during DAIR for FRI or revision surgery may enhance pathogen identification; this should be studied further. We recommend validation and a consideration of implementing SFC in future FRI consensus guidelines.

## Data Availability

The original contributions presented in the study are included in the article; further inquiries can be directed to the corresponding author.
